# Remission by composite scores in rheumatoid arthritis: are ankles and feet important?

**DOI:** 10.1186/ar2270

**Published:** 2007-07-27

**Authors:** Theresa Kapral, Florian Dernoschnig, Klaus P Machold, Tanja Stamm, Monika Schoels, Josef S Smolen, Daniel Aletaha

**Affiliations:** 1 Department of Rheumatology, Medical University of Vienna, Waehringer Guertel 18-20, 1090 Vienna, Austria; 2 2nd Department of Medicine, Hietzing Hospital, Wolkersbergengasse 1, 1130 Vienna, Austria

## Abstract

Current treatment strategies aim to achieve clinical remission in order to prevent the long-term consequences of rheumatoid arthritis (RA). Several composite indices are available to assess remission. All of them include joint counts as the assessment of the major 'organ' involved in RA, but some employ reduced joint counts, such as the 28-joint count, which excludes ankles and feet.

The aim of the present study was to determine the relevance of excluding joints of the ankles and feet in the assessment of RA disease activity and remission. Using a longitudinal observational RA dataset, we analyzed 767 patients (80% female, 60% rheumatoid factor-positive), for whom joint counts had been recorded at 2,754 visits. We determined the number of affected joints by the 28-JC and the 32-JC, the latter including ankles and combined metatarso-phalangeal joints (as a block on each side).

Several findings were supportive of the validity of the 28-joint count: (a) Absence of joint swelling on the 28-joint scale had a specificity of 98.1% and a positive predictive value (PPV) of 94.1% for the absence of swelling also on the 32-joint scale. For absence of tender joints, the specificity and PPV were 96.1% and 91.7%, respectively. (b) Patients with swollen or tender joints in the 32-JC, despite no joint activity in the 28-JC, were clearly different with regard to other disease activity measures. In particular, the patient global assessment of disease activity was higher in these individuals. Thus, the difference in the joint count was not relevant for composite disease activity assessment. (c) The disease activity score based on 28 joints (DAS28) may reach levels higher than 2.6 in patients with feet swelling since these patients often have other findings that raise DAS28. (d) The frequency of remission did not change when the 28-JC was replaced by 32-JC in the composite indices. (e) The changes in joint activity over time were almost identical in longitudinal analysis.

The assessment of the ankles and feet is an important part in the clinical evaluation of patients with RA. However, reduced joint counts are appropriate and valid tools for formal disease activity assessment, such as done in composite indices.

## Introduction

The ultimate therapeutic goals in rheumatoid arthritis (RA) are the prevention of joint destruction and the restoration of functional abilities. Since progression of joint damage stops or becomes minimal in situations of clinical remission [1], controlling disease activity, and ideally achieving remission, has become an important therapeutic goal [2]. New therapies and the novel therapeutic strategies that evolved during the past decade have moved this aim into reach for a considerable number of patients with RA [3].

In the evaluation of patients with arthritis, evaluating articular involvement corresponds to evaluating the 'organ' involved in the disease. Joints are usually assessed for tenderness and swelling, and joint counts are predictive of radiographic changes as well as of long-term morbidity and mortality in RA [4-7]. Joint counts are commonly used to evaluate joint involvement and are therefore an indispensable component of formal disease activity assessment using composite indices. Although principally in clinical practice all relevant joints should be evaluated in patients with RA, various joint count scales have been developed to reduce the burden of formal joint count assessment, which is important in longitudinal follow-up of patients with RA and objective evaluation of treatment response. In these scales, the numbers of examined joints range from 16 to 74 [8]. For clinical practice and in several recent clinical trials [9-11], the reduced 28-joint counts have been used frequently for feasibility and logistic reasons and correlate well with extended joint counts [12,13]. However, it has been a matter of debate whether the exclusion of ankles and feet, as in the 28-JC, may put at risk the definition of remission [14]. In the present study, we aimed to compare the 28-JC with the 32-JC (additionally assessing ankle and metatarso-phalangeal [MTP] joints) in the context of evaluating joint remission and remission of disease activity by composite indices.

## Materials and methods

### Patients

We studied 767 consecutive patients with RA [15] who had received at least one course of disease-modifying antirheumatic drug (DMARD) therapy and had at least one follow-up visit after initiation of DMARD therapy with complete data recording as required for this investigation. All patients were followed in the rheumatology outpatient clinics at the Vienna General Hospital and the Hietzing Hospital (Vienna). Both clinics are specialized referral centers where patients are usually seen every 3 months by rheumatologists or physicians in rheumatology training. Since 1997, visits of patients with RA have been documented prospectively in a longitudinal observational RA dataset. Data quality in this CARAbase ('CAre of RA database') is ensured by periodical updates of missing data by data entry personnel, as previously described [16-18]. Patients gave their consent for anonymous analysis of the obtained clinical data.

### Definition and identification of visits

We identified all outpatient visits in the dataset with complete documentation of all disease activity variables that are routinely assessed, including 28- and 32-joint counts. All other visits were excluded for this analysis. The 28-joint count [12] comprises the shoulder (*n *= 2), elbow (*n *= 2), wrist (*n *= 2), knee (*n *= 2), metacarpophalangeal (*n *= 10), and proximal interphalangeal (*n *= 8) joints and the interphalangeal joints of the thumbs (*n *= 2). The 32-joint count additionally evaluates the ankle joints (*n *= 2) and all MTP joints assessed as one group on each side (*n *= 2). Joint assessment had been performed by trained health professionals who were unaware of the purpose of the study.

The 767 patients completed a total of 2,754 visits, in which joint counts on both scales were documented. Additional data comprised C-reactive protein (CRP) and erythrocyte sedimentation rate, measures of pain, patient and physician global assessments of disease activity by 100-mm visual analog scales, and the health assessment questionnaire disability index [19]. These data allowed calculation of the disease activity score based on 28 joints (DAS28) [20] and the simplified disease activity index (SDAI) [21]. To preserve the independence of observations, which is a prerequisite for most statistical analyses, we used only the first visit of each patient documented in the dataset for most analyses. This first documented visit was not necessarily the patient's first visit at the clinics.

### Statistical analyses

We first calculated the specificity and positive predictive value of no 'joint activity' (that is, JC = 0) by the 28-joint count ('28-joint count remission', 28JC^-^) for no activity also by the 32-joint count (32JC^-^). 'Joint activity' refers to swollen joints or tender joints, as appropriate. In this regard, the term 'residual' tenderness/swelling refers to the number of the four joint areas of the feet in patients without any active joint by the 28-JC.

We then tested whether levels of disease activity, as evaluated by composite scores, were different between patients with no active joint by the 32-joint count (32JC^-^) and those with no active joint by the 28JC but active joints by the 32-joint scale (28JC^-^/32JC^+^). Since the 28JC^-^/32JC^+ ^patients comprised only a small number, we used all observations of patients in the dataset (*n *= 2,754) instead of only the first fully documented visit of each patient. We tested for differences in the DAS28 and SDAI levels, respectively, between the two groups and accounted for potential multiple observations per patient by employing a linear mixed model. This model used the 32JC^- ^versus 28JC^-^/32JC^+ ^status as a fixed factor.

Since remission should represent a well-defined and specific state irrespective of the joint count employed, we applied the remission criteria of the DAS28 (less than 2.6) and the SDAI (less than or equal to 3.3) [22,23] and compared the residual joint activity by the 28-JC and 32-JC.

In another cross-sectional analysis, we calculated the DAS28 and SDAI using the 32-JC instead of the 28-JC ('DAS32' and 'SDAI32'). Although these indices are not validated for use with a 32-JC, this exploratory comparison allowed assessment of the impact of the potentially higher number of swollen and tender joints on these common instruments of overall disease activity. We compared the impact on remission frequencies by these two calculations using the χ^2 ^statistics.

In a final, longitudinal analysis, we looked at the responses of both joint counts over two subsequent visits in these patients. We correlated the changes observed in the 28-JC scales with those seen in the 32-JC scales using the Pearson correlation. All statistical analyses were carried out using SPSS (Statistical Package for the Social Sciences) version 12.0 (SPSS Inc., Chicago, IL, USA).

## Results

### Patients

The characteristics of the 767 patients are presented in Table 1. Patients had a mean (± standard deviation [SD]) age of 54.1 (± 14.9) years, 79.9% were female, and 55.3% were rheumatoid factor-positive. Their mean (± SD) disease duration at database entry was 8.1 (± 10.6) years. For these patients, we identified 2,754 outpatient visits in which complete records of 28- and 32-joint counts were available.

**Table 1:** Characteristics of 767 patients

Patients	
Age in years (mean ± SD)	54.1 ± 14.9
Female gender	79.9%
Rheumatoid factor-positive	55.3%
Disease duration at baseline in years (mean ± SD)	8.1 ± 10.6
Duration of follow-up in years (mean ± SD; range)	5.1 ± 1.4; 6.8
Disease activity characteristics, median (quartiles)	
Swollen joint count (0–28)	3 (1; 7)
Tender joint count (0–28)	2 (0; 6)
Erythrocyte sedimentation rate in millimeters (normal <20)	23 (14; 55)
C-reactive protein in milligrams per deciliter (normal <1.0)	1.1 (0.5; 2.7)
Patient assessment of pain in millimeters (0–100)	37 (19; 53)
Patient global assessment of activity in millimeters (0–100)	37 (18; 58)
Physician global assessment of activity in millimeters (0–100)	34 (19; 49)
Health Assessment Questionnaire (0–3)	0.875 (0.25; 1.5)

SD, standard deviation.

### Reduced joint counts are specific in assessing absence of joint activity

**Figure 1:**
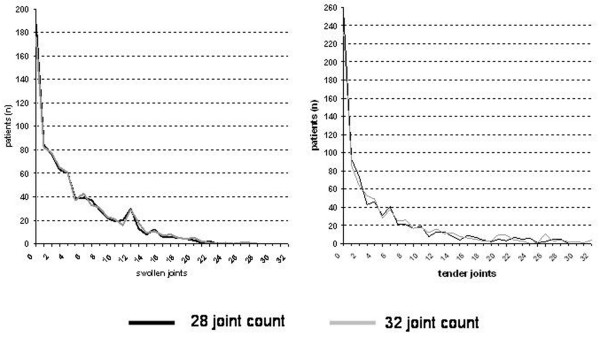
Frequency of joint involvement. Twenty-eight- and 32-joint count scales provided similar results in 98.6% for the swollen joint assessment **(a) **and in 97.3% for the tender joint assessment **(b)**.

When we evaluated patients with no swollen joints according to the 28-JC, the 28- and 32-joint count scales provided identical results in 98.6% for the swollen joint assessment and in 97.3% for the tender joint assessment (Figure 1).

The 28-JCs were 0 in 187 patients (Tables 2 and 3, '28-JC remission'), and only 11 (5.9%) of them had swollen ankle and/or MTP joints. No swelling of any joint on the 28-joint count scale (Table 2: 28-JC remission 'Yes') had a specificity of 98.1% and a positive predictive value of 94.1% for the absence of swelling also on the 32-JC scale (Table 2). Tender joint counts were 0 by the 28-JC in 254 patients, and only 21 (8.3%) of them had tender ankle and/or MTP joints. No tenderness of any joint on the 28-joint count scale had a specificity of 96.1% and a positive predictive value of 91.7% for the absence of joint tenderness by the 32-joint scale (Table 3). Thus, the 28-JC is highly specific also in regard to absence of activity in the lower extremity joints.

**Table 2:** Frequencies of joint remission by different scales and by swelling

		32-JC remission		
		
		Yes	No	
28-JC remission	Yes	176	11	187
	No	0	580	580
		176	591	

28-JC, 28 joint count; 32-JC, 32 joint count.

**Table 3:** Frequencies of joint remission by different scales and by tenderness

		32-JC remission		
		
		Yes	No	
28-JC remission	Yes	233	21	254
	No	0	513	513
		233	534	

28-JC, 28 joint count; 32-JC, 32 joint count.

### Patients with residual joint activity by extensive joint counts are also different in most other disease activity measures

In this analysis, we used multiple observations per patient (2,754 visits) and a mixed model. We compared DAS28 and SDAI levels of 32JC^- ^patients with 28JC^-^/32JC^+ ^patients. No swollen or tender joints by the 32-JC were observed in 760 and 1,120 visits, respectively, whereas 38 (4.8%) and 102 (8.3%) visits were 28JC^-^/32JC^+^. Linear mixed models accounting for repeated measurements within patients showed significant differences for SDAI as well as patient pain and patient global assessments between the 32JC^- ^and 28JC^-^/32JC^+ ^groups (Table 4). DAS28 was significantly different only for 32JC^- ^versus 28JC^-^/32JC^+ ^tender joints (Table 4).

**Table 4:** Disease activity in patients with complete and incomplete joint remission

	Swollen joints			Tender joints		
	32JC^- ^(*n *= 760)	28JC^-^/32JC^+ ^(*n *= 38)	*p*	32JC^- ^(*n *= 1,120)	28JC^-^/32JC^+ ^(*n *= 102)	*p*

SDAI	6.45	8.93	0.03	7.95	10.91	0.00
DAS28	2.65	2.92	0.11	2.65	2.97	0.00
C-reactive protein (mg/dl)	1.25	1.43	0.56	1.38	1.52	0.42
Pain (mm VAS)	23	36	0.00	20	34	0.00
Patient global (mm VAS)	22	39	0.00	21	35	0.00
Physician global (mm VAS)	17	19	0.51	12	25	0.00

Complete joint remission refers to the situation with no joint activity by the 32-joint count (32JC^-^), while incomplete remission refers to patients with no joint activity by the 28, but active joints by the 32-joint scale (28JC^-^/32JC^+^). DAS28, disease activity score based on 28 joints; SDAI, simplified disease activity index; VAS, visual analog scale.

Among the 38 visits with 28JC^-^/32JC^+ ^swollen joint counts, only 13 (34%) had a DAS28 of less than 2.6 and only 3 (8%) had an SDAI of less than or equal to 3.3; among the 102 visits with 28JC^-^/32JC^+ ^tender joint counts, these numbers amounted to 32 (31%) and 8 (8%), respectively. This indicates that the majority of patients with residual joint activity in the feet would not meet standard remission criteria based on their overall disease activity. Furthermore, the mean patient global assessments among visits with residual 32-JC joint involvement were higher than the cut-point for SDAI remission (3.9 cm for those with residual swollen joints and 3.5 cm for those with residual tender joints; Table 4) already before any of the other variables (such as CRP) were accounted for. Thus, only very few patients with presence of swollen or tender joints by the 32-JC fulfilled SDAI remission criteria. Thus, patients with joint activity by the 32-JC despite remission by the 28-JC were clearly different in regard to other disease activity measures. Thus, the difference in the joint count does not seem to be relevant for composite disease activity assessment.

### Different scales provide similar joint counts in patients fulfilling remission criteria

**Figure 2:**
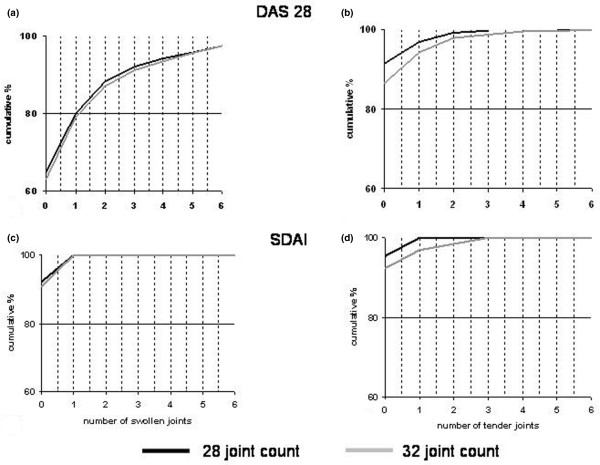
Joint counts in clinical remission. Cumulative distributions of observed residual swollen and tender joints in patients with DAS28 (disease activity score based on 28 joints) remission or SDAI remission. **(a) **residual swollen joints in DAS28 remission; **(b) **residual tender joints in DAS28 remission; **(c) **residual swollen joints in SDAI remission; **(d) **residual tender joints in SDAI remission.

In 126 (16.4%) of the 767 patients, DAS28 remission (DAS28 of less than 2.6) was observed. Among those observations, the 32-joint count identified presence of swollen ankle or foot joints in only 2.4%. The proportion of patients with presence of tender ankle or foot joints in DAS28 remission was slightly higher (6.3%). The cumulative distributions of observed presence of total swollen and tender joints in patients with DAS28 remission are presented in Figure 2a and 2b. The maximum number of total swollen joints in DAS28 remission was 16. When assessing SDAI remission, we found only 65 patients (8.5%) fulfilling the criteria (SDAI of less than or equal to 3.3). Among these patients, residual swollen ankle or foot joints in the 32-joint count were found in only 1.5% and residual tender ankle or foot joints in 3.1%; the cumulative proportions are presented in Figure 2c and 2d. Thus, in patients who achieve a state of remission, the frequency of observable joint activity is comparable between the two scales.

### Remission frequency is not affected if more extensive joint counts are used in composite measures of disease activity

In an exploratory analysis, we employed the 32-joint counts to calculate disease activity indices using the DAS28 and SDAI formulae and defined remission by the traditional DAS28 and SDAI cut-points. The number of patients in remission ('DAS32' of less than 2.6 and 'SDAI32' of less than or equal to 3.3) remained very similar to the analyses employing the 28-joint counts: 120 patients (15.6%) fulfilled a 'DAS32' of less than 2.6 compared to 127 (16.5%) by the DAS28, and 63 (8.2%) met 'SDAI32' of less than or equal to 3.3 compared to 65 patients (8.5%) in remission by the SDAI using the 28-JCs (Pearson correlation: *p *value not significant). Thus, assessment of ankles and MTPs had few implications for establishing the frequency of remissions. Thus, the frequency of remission did not change when the 28-JCs were replaced by 32-JC in the composite indices.

### Longitudinal changes in joint activity are similar between different joint count methods

**Figure 3:**
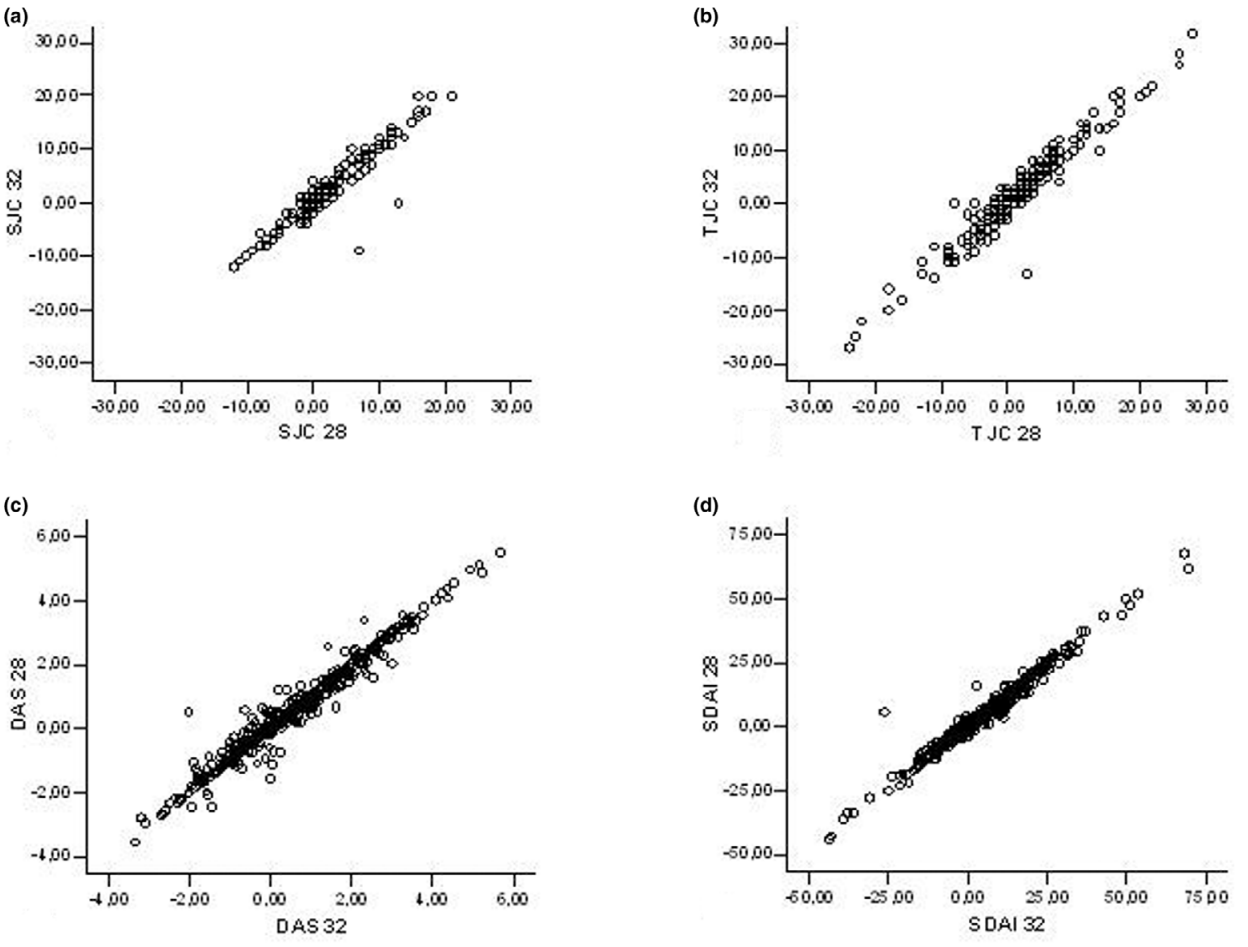
Longitudinal response of joint counts and composite indices. Pearson correlation coefficient revealed a strong positive association between swollen **(a) **and tender **(b) **28-joint counts and 32-joint counts and an almost perfect linear relationship between DAS28 and 'DAS32' **(c) **or SDAI and 'SDAI32' **(d)**.

To evaluate the differences in longitudinal assessment of joint activity, we correlated the changes observed in the 28-joint counts with the changes observed in the 32-joint counts at two subsequent visits. The Pearson correlation coefficient was between 0.96 and 1.0, revealing a strong positive association between the two joint counts and an almost perfect linear relationship between DAS28 and 'DAS32' or SDAI and 'SDAI32', respectively (Figure 3a–d). Thus, the changes in joint activity over time were almost identical in longitudinal analysis.

## Discussion

The joints are the major 'organ' involved in RA. Formal evaluation of joint activity, therefore, is a prerequisite of disease activity assessment in RA. In this study, we showed that the assessment of joint activity in the feet, beyond assessment of joint activity by the 28-joint count, does not convey significant added value in the evaluation of disease activity. This conclusion is a consequence of the less frequent involvement of ankle and foot joints than joints of the upper extremities [13] and the rare occurrence of isolated foot involvement in remission states as revealed here. Moreover, changes over time are not significantly different between the 28- and the 32-joint counts or between disease activity indices that employ those joint counts.

Outcome measurement in RA is rich in different scales to assess joint activity, leading to a tension between comprehensiveness and thus the ultimate assurance of sensitivity (to leave 'no joint undetected') and feasibility. The need for one or the other is usually also a function of the setting in which disease activity assessment is performed. Whereas in clinical trials the highest degree of sensitivity in detection and responsiveness of active joints might be a predominant goal, it will be the least time-consuming method in clinical practice [24]. At a time when clinical remission has become an achievable goal, another issue is of importance: the *specificity *of the term 'remission'. In this regard, our study showed no relevant differences between the 28- and the 32-joint count scales, with positive predictive values of 28-joint remission above 95% in our cohort.

The reduced 28-joint count has become widely used in recent years. Its simplicity as a mere joint count or in the context of DASs has also led to acceptance in several of the contemporary trials [11,25], but it is especially employed in observational studies in which assessment times become a logistic challenge as patients are often seen in routine clinical practice. However, its validity with respect to classification of remission has been challenged recently, and it was suggested that no patient should be classified as being in remission without a full joint assessment [14]. Although the 32-joint count (in contrast to 28- and 36-joint counts [26]) has not been formally evaluated, Ritchie and colleagues [27] have already shown (40 years ago) the validity of evaluating all metacarpo-phalangeal joints together. This has been done here for the MTP joints, which are combined in the 32-joint count into a single joint. The assessment of all MTPs together appears sufficient to identify tenderness and swelling generally, although detailed MTP joint counts cannot be derived.

Looking at differences between 28- and 32-joint counts in our prospective observational dataset, we found concordance rates as well as sensitivity of 28-joint counts in the order of 95% and above using the 32-joint count as a gold standard. Feet and ankles were only rarely involved if the 28-joint counts were 0. Conversely, a few patients had up to 2 joint regions involved in these situations (swollen: *n *= 5, 0.6%; tender: *n *= 10, 1.3%). In those few individuals, our study is limited with respect to the exact number of involved joints since we counted the MTPs on each side as one joint only. Fewer than 6% of observations among patients with no joints involved by the 28-joint count had evidence of residual swollen joints, and less than 9% had evidence of residual tender ankle or foot joints by the more extended joint count. Interestingly, while under such circumstances the composite indices showed significantly higher scores compared to patients in whom no joint of the 32-JCs was involved, the vast majority of these patients would not fulfill SDAI remission criteria: already without accounting for any of the other SDAI components, the mean patient global assessment amounted to higher values than would be compatible with the definition of remission. Thus, despite full reversal of joint activity by the 28-JCs, patients with residual involvement of the ankles and feet had other complaints of sufficient degree to prevent their classification into remission. Thus, by indices that employ the 28-JC, there is only a very small number of patients in remission who have joints involved that are not contained in the 28-JC, indicating that omitting ankle and foot joint assessment from such indices does not significantly jeopardize the definition of remission. This finding also reveals that information on remission by composite scores employing 28-JCs is rarely erroneous. This is primarily true for information provided by the SDAI. However, the DAS28 does not appear to lead to erroneous conclusions due to omission of more comprehensive JCs. On the other hand, in the present study, we found up to 16 swollen joints in DAS28 remission (not yet accounting for ankles and MTPs). This residual disease activity seen in DAS28 remission is due to the construction of this score, which has also been stated by several authors previously; up to 20% of patients in remission defined by DAS28 may have 2 or more residual swollen joints [23,28,29]. A similar result has been obtained for the traditional DAS, which employs extended JCs [29].

It appears less important in the definition of remission whether a few more joints of the feet are assessed than whether remission criteria cut-points are sufficiently stringent. The data from the literature and from this study together suggest that a DAS28 level of less than 2.6 is not sufficiently specific to serve as a cut-point for remission whereas the SDAI cut-point of less than or equal to 3.3 does appear appropriate. Furthermore, to eliminate any principal weakness of the DAS28 remission cut-point, we performed our analyses also using the SDAI remission criteria with the 32-JC rather than the 28-JC in the formula; even these conditions changed the proportion of patients in remission only minimally.

Finally, an important clinical consideration should be discussed. The mere fact that ankles and feet have been excluded in the context of certain composite scores does not justify their omission in the evaluation and management of individual patients with RA. In contrast, since their involvement is common and they bear highly important functional roles, ankle and MTP joints have been included in our routine clinical assessments of patients with RA via the 32-joint counts that are recorded in our database.

## Conclusion

Our data provide evidence that while providing useful and important clinical information, the inclusion of ankles and feet only rarely influences the definition of overall disease activity status, especially the presence or absence of remission. Composite indices based on 28-JCs are valid for the assessment of disease activity.

## Abbreviations

28-JC = 28-joint count; 32-JC = 32-joint count; CRP = C-reactive protein; DAS = disease activity score; DAS28 = disease activity score based on 28 joints; DMARD = disease-modifying antirheumatic drug; JC = joint count; MTP = metatarso-phalangeal; RA = rheumatoid arthritis; SD = standard deviation; SDAI = simplified disease activity index.

## Competing interests

The authors declare that they have no competing interests.

## Authors' contributions

TK performed study design, data analysis, manuscript drafting, and data acquisition. JSS and DA performed study design, data analysis, and manuscript drafting. FD, TS, KPM, and MS performed data acquisition. All authors read and approved the final manuscript.

## References

[B1] Molenaar ET, Voskuyl AE, Dinant HJ, Bezemer PD, Boers M, Dijkmans BA (2004). Progression of radiologic damage in patients with rheumatoid arthritis in clinical remission. Arthritis Rheum.

[B2] Emery P, Salmon M (1995). Early rheumatoid arthritis: time to aim for remission?. Ann Rheum Dis.

[B3] O'Dell JR (2004). Therapeutic strategies for rheumatoid arthritis. N Engl J Med.

[B4] Sharp JT, Lidsky MD, Duffy J (1982). Clinical responses during gold therapy for rheumatoid arthritis. Changes in synovitis, radiologically detectable erosive lesions, serum proteins, and serologic abnormalities. Arthritis Rheum.

[B5] Scott DL, Coulton BL, Bacon PA, Popert AJ (1985). Methods of x-ray assessment in rheumatoid arthritis: a re-evaluation. Br J Rheumatol.

[B6] Fuchs HA, Callahan LF, Kaye JJ, Brooks RH, Nance EP, Pincus T (1988). Radiographic and joint count findings of the hand in rheumatoid arthritis. Related and unrelated findings. Arthritis Rheum.

[B7] Pincus T, Callahan LF (1992). Quantitative measures to assess, monitor and predict morbidity and mortality in rheumatoid arthritis. Baillieres Clin Rheumatol.

[B8] Aletaha D, Smolen JS (2006). The definition and measurement of disease modification in inflammatory rheumatic diseases. Rheum Dis Clin North Am.

[B9] Dougados M, Emery P, Lemmel EM, Zerbini CA, Brin S, van Riel P (2005). When a DMARD fails, should patients switch to sulfasalazine or add sulfasalazine to continuing leflunomide?. Ann Rheum Dis.

[B10] Flendrie M, Creemers MC, Welsing PM, van Riel PL (2005). The influence of previous and concomitant leflunomide on the efficacy and safety of infliximab therapy in patients with rheumatoid arthritis; a longitudinal observational study. Rheumatology (Oxford).

[B11] Emery P, Breedveld FC, Lemmel EM, Kaltwasser JP, Dawes PT, Gömör B, Van Den Bosch F, Nordström D, Bjorneboe O, Dahl R (2000). A comparison of the efficacy and safety of leflunomide and methotrexate for the treatment of rheumatoid arthritis. Rheumatology (Oxford).

[B12] Fuchs HA, Pincus T (1994). Reduced joint counts in controlled clinical trials in rheumatoid arthritis. Arthritis Rheum.

[B13] Smolen JS, Breedveld FC, Eberl G, Jones I, Leeming M, Wylie GL, Kirkpatrick J (1995). Validity and reliability of the twenty-eight-joint count for the assessment of rheumatoid arthritis activity. Arthritis Rheum.

[B14] Landewé R, van der Heijde D, van der Linden S, Boers M (2006). Twenty-eight-joint counts invalidate the DAS28 remission definition owing to the omission of the lower extremity joints: a comparison with the original DAS remission. Ann Rheum Dis.

[B15] Arnett FC, Edworthy SM, Bloch DA, McShane DJ, Fries JF, Cooper NS, Healey LA, Kaplan SR, Liang MH, Luthra HS (1988). The American Rheumatism Association 1987 revised criteria for the classification of rheumatoid arthritis. Arthritis Rheum.

[B16] Aletaha D, Smolen JS (2002). The rheumatoid arthritis patient in the clinic: comparing more than 1,300 consecutive DMARD courses. Rheumatology (Oxford).

[B17] Aletaha D, Smolen JS (2002). Effectiveness profiles and dose dependent retention of traditional disease modifying antirheumatic drugs for rheumatoid arthritis. An observational study. J Rheumatol.

[B18] Aletaha D, Smolen JS (2002). Laboratory testing in rheumatoid arthritis patients taking disease-modifying antirheumatic drugs: clinical evaluation and cost analysis. Arthritis Rheum.

[B19] Fries JF, Spitz P, Kraines RG, Holman HR (1980). Measurement of patient outcome in arthritis. Arthritis Rheum.

[B20] Prevoo ML, van 't Hof MA, Kuper HH, van Leeuwen MA, van de Putte LB, van Riel PL (1995). Modified disease activity scores that include twenty-eight-joint counts. Development and validation in a prospective longitudinal study of patients with rheumatoid arthritis. Arthritis Rheum.

[B21] Smolen JS, Breedveld FC, Schiff MH, Kalden JR, Emery P, Eberl G, van Riel PL, Tugwell P (2003). A simplified disease activity index for rheumatoid arthritis for use in clinical practice. Rheumatology (Oxford).

[B22] Fransen J, Creemers MC, van Riel PL (2004). Remission in rheumatoid arthritis: agreement of the disease activity score (DAS28) with the ARA preliminary remission criteria. Rheumatology (Oxford).

[B23] Aletaha D, Ward MM, Machold KP, Nell VP, Stamm T, Smolen JS (2005). Remission and active disease in rheumatoid arthritis: defining criteria for disease activity states. Arthritis Rheum.

[B24] Aletaha D, Eberl G, Nell VP, Machold KP, Smolen JS (2004). Attitudes to early rheumatoid arthritis: changing patterns. Results of a survey. Ann Rheum Dis.

[B25] Smolen JS, Kalden JR, Scott DL, Rozman B, Kvien TK, Larsen A, Loew-Friedrich I, Oed C, Rosenburg R (1999). Efficacy and safety of leflunomide compared with placebo and sulphasalazine in active rheumatoid arthritis: a double-blind, randomised, multicentre trial. European Leflunomide Study Group. Lancet.

[B26] Egger MJ, Huth DA, Ward JR, Reading JC, Williams HJ (1985). Reduced joint count indices in the evaluation of rheumatoid arthritis. Arthritis Rheum.

[B27] Ritchie DM, Boyle JA, McInnes JM, Jasani MK, Dalakos TG, Grieveson P, Buchanan WW (1968). Clinical studies with an articular index for the assessment of joint tenderness in patients with rheumatoid arthritis. Q J Med.

[B28] Mäkinen H, Kautiainen H, Hannonen P, Sokka T (2005). Is DAS28 an appropriate tool to assess remission in rheumatoid arthritis?. Ann Rheum Dis.

[B29] van der Heijde D, Klareskog L, Boers M, Landewé R, Codreanu C, Bolosiu HD, Pedersen R, Fatenejad S, TEMPO Investigators (2005). Comparison of different definitions to classify remission and sustained remission: 1 year TEMPO results. Ann Rheum Dis.

